# The function of the complement system remains fully intact throughout the course of allogeneic stem cell transplantation

**DOI:** 10.3389/fimmu.2024.1422370

**Published:** 2024-06-13

**Authors:** Beatrice Fageräng, Leon Cyranka, Camilla Schjalm, Karin Ekholt McAdam, Carina Sandem Larsen, Julia Heinzelbecker, Tobias Gedde-Dahl, Reinhard Würzner, Terje Espevik, Geir Erland Tjønnfjord, Peter Garred, Andreas Barratt-Due, Tor Henrik Anderson Tvedt, Tom Eirik Mollnes

**Affiliations:** ^1^ Department of Immunology, Oslo University Hospital and University of Oslo, Oslo, Norway; ^2^ Department of Clinical Immunology, Laboratory of Molecular Medicine, Copenhagen University Hospital - Rigshospitalet, Copenhagen, Denmark; ^3^ Department of Hematology, Oslo University Hospital, Oslo, Norway; ^4^ Institute of Clinical Medicine, University of Oslo, Oslo, Norway; ^5^ Institute of Hygiene and Medical Microbiology, Medical University of Innsbruck, Innsbruck, Austria; ^6^ Department of Clinical and Molecular Medicine, Norwegian University of Science and Technology, Trondheim, Norway; ^7^ Division of Emergencies and Critical Care, Oslo University Hospital, Oslo, Norway; ^8^ Research Laboratory, Nordland Hospital, Bodø, Norway

**Keywords:** HSCT, immunosuppression, innate immunity, complement, cytokines

## Abstract

**Introduction:**

Hematopoietic stem cell transplantation (HSCT) is associated with immune complications and endothelial dysfunction due to intricate donor-recipient interactions, conditioning regimens, and inflammatory responses.

**Methods:**

This study investigated the role of the complement system during HSCT and its interaction with the cytokine network. Seventeen acute myeloid leukemia patients undergoing HSCT were monitored, including blood sampling from the start of the conditioning regimen until four weeks post-transplant. Clinical follow-up was 200 days.

**Results:**

Total complement functional activity was measured by WIELISA and the degree of complement activation by ELISA measurement of sC5b-9. Cytokine release was measured using a 27-multiplex immuno-assay. At all time-points during HSCT complement functional activity remained comparable to healthy controls. Complement activation was continuously stable except for two patients demonstrating increased activation, consistent with severe endotheliopathy and infections. *In vitro* experiments with post-HSCT whole blood challenged with *Escherichia coli*, revealed a hyperinflammatory cytokine response with increased TNF, IL-1β, IL-6 and IL-8 formation. Complement C3 inhibition markedly reduced the cytokine response induced by *Staphylococcus aureus*, *Aspergillus fumigatus*, and cholesterol crystals.

**Discussion:**

In conclusion, HSCT patients generally retained a fully functional complement system, whereas activation occurred in patients with severe complications. The complement-cytokine interaction indicates the potential for new complement-targeting therapeutic strategies in HSCT.

## Introduction

Allogeneic hematopoietic stem cell transplantation (HSCT) is a highly effective treatment for various hematologic disorders (1). Despite significant advancement and improved survival rates, the transplant procedure remains linked to a considerable incidence of treatment-related morbidity and mortality (2). This association is attributed to immune-mediated complications, notably graft-versus-host disease (GVHD) (3), as well as infectious complications and endothelial syndromes including transplant-associated thrombotic microangiopathy (TA-TMA) (4, 5).

The early immunological responses following HSCT are typically split into three overlapping phases (6). The first phase is characterized by an inflammatory milieu with increased pathogen- and damage-associated molecular pattern (PAMP/DAMP) levels and inflammatory mediators induced by the conditioning regimen (3, 6). The proinflammatory state leads to the activation of antigen-presenting host cells, initiating the activation of donor T-cells (7), neovascularization and endothelial dysfunction (8, 9). A third, effector phase, GVHD, may occur upon sufficient alloreactivity. Tissue damage is caused by direct CD8-mediated cytotoxicity and release of inflammatory mediators (3, 10).

Much research has focused on the cellular immune system during HSCT (11), whereas less is known about the humoral response, such as the complement system. The complement system is an innate immunity sensor, functioning as an upstream recognition alarm system that discerns threats from exogenous and endogenous sources (12). The specific functions of the complement system include microbial defense by phagocytosis, with the opsonization of the microbe by iC3b binding to the CR3 receptor. Other main effector functions are mediated through the formation of the highly potent C5a molecule, which induces inflammation, and the lytic terminal C5b-9 complex, which can penetrate bacterial and cellular membranes (12). Importantly, inappropriate or excessive activation of complement can damage the host severely.

The complement system is highly relevant for HSCT, as these patients are at higher risk for infections (5) and endothelial disorders (13), which are linked to complement function. TA-TMA is characterized by endothelial cell activation, microvascular hemolytic anemia, and complement dysregulation (13, 14). Several studies have shown that increased complement activation as measured by the soluble C5b-9 complex (sC5b-9) predicts the development of TA-TMA (13, 15–17). Therapeutic inhibition targeted at complement component C5, preventing C5a and C5b-9 formation, seems promising as reported in a large pediatric cohort with TA-TMA (18). Furthermore, a multi-center study has previously indicated a correlation between complement activation and outcome after HSCT (19). In this study we aimed to investigate the innate immune profile, including neutrophil and platelet activation, the cytokine network, and, in particular, the function and activation of complement in HSCT.

## Methods

### Study design, patients, transplantation procedure and acquisition of blood samples

Acute myeloid leukemia (AML) patients (n=17) undergoing allogeneic HSCT were recruited by the Hematology Department at Oslo University Hospital.

A summary of the various conditioning regimens is provided in **Supplementary Table 1**. All patients were treated with cyclosporine-based GVHD prophylaxis which consisted of post-transplant cyclophosphamide, mycophenolate mofetil, and cyclosporin (starting on day +5) for haploidentical donors or donors with HLA-A or HLA-B mismatches, or anti-thymocyte globulin, methotrexate, and cyclosporine (starting on day -1) for other donors. All patients received antifungal prophylaxis with posaconazole from day 0 until engraftment, and fluconazole from engraftment until day +75 post-transplant. Antibacterial prophylaxis was not routinely administered, and growth factors were only administered in patients with haploidentical donors. All patients received ursodeoxycholic acid from the start of conditioning regimens until day 100. The total observation time for the patients was 200 days post-transplant.

Samples were obtained six days before transplantation (day -6), one day before transplantation (day -1), one day after transplantation (day +1), and weekly following transplantation (day +7, day +14, day +21 and day +28). The HSCT transplantation process commences with six days of chemotherapy; the day -6 sample was obtained before the first chemotherapy dose and was considered as baseline sample. The day -6 sample was compared with samples from healthy median age and gender-matched controls (n= 10) recruited during the same period. There were only nine samples in the whole material that were not obtained; this due to administrative difficulties.

Three tubes of blood (4.5 mL) were drawn: 1) Vacuette K_2_EDTA tubes (Greiner Bio-One, Kremsmünster, Austria), 2) silica-clotted tubes for preparing of serum (Greiner Bio-One), and 3) endotoxin-free NUNC tubes (Thermo Fischer Scientific, Roskilde, Denmark) prefilled with the specific thrombin inhibitor lepirudin. All tubes were stored in an ice-water bath and processed within two hours after collection. Immediately after receiving the samples, EDTA plasma was prepared by centrifugation (3000 × *g*, 15 min, at 4°C). Blood for serum preparation was incubated at room temperature until clotting and centrifuged as for the EDTA plasma. Plasma and serum were aliquoted and immediately stored at -80°C.

### The lepirudin whole blood model

Whole blood was sampled into sterile NUNC cryo tubes containing lepirudin (Refludan® Pharmion, Copenhagen, Denmark) at a final concentration of 50 µg/mL for anticoagulation. The whole blood model was performed as previously described (20). Lepirudin blood was incubated with PBS (negative control), 10^7^ *E. coli*/mL, 10^7.5^ *E. coli*/mL, 10^7^ *S. aureus*/mL, 10^7^ *C. albicans*/mL, 10^7^ *A. fumigatus*/mL, or 1 mg/mL cholesterol crystals for 0, 30, and 120 min. Complement activation was stopped, by adding 20 mM of EDTA (final concentration) at the indicated timepoints. Whole blood samples were centrifuged at 3000 x *g*, 15 min, at 4°C to obtain plasma, which was stored at -80°C until further analysis. Incubation with microbes was performed with heat-inactivated *E. coli* (ATCC-33572, American Type Culture Collection, Manassas, VA), heat-inactivated *S. aureus* (Cowan, ATCC-12598, American Type Culture Collection), heat inactivated *C. albicans* (SC5314, ATCC MYA-287, American Type Culture Collection), and heat-inactivated *A. fumigatus* (clinically isolated strain 6881 cultivated at Copenhagen University Hospital - Rigshospitalet, Copenhagen). Cholesterol crystals were prepared as described by Samstad et al. (21). Inhibition of the complement system was performed using 10 μM (final concentration) of the C3-inhibitor Cp40 (compstatin) (22).

### Complement function and activation

Complement function was measured in fresh-frozen serum in the WIESLAB® Total Complement System Screen kit (SVAR, Malmö, Sweden), an enzyme immunoassay previously described (23). Results are given in percent of a normal serum pool defined to contain 100% complement function. Complement activation was measured in plasma by an ELISA detecting the sC5b-9, as originally described (24), and performed as later modified (25). sC5b-9 was detected by the mAb aE11 reacting with a neoepitope expressed in C9 when incorporated into C5b-9 and not expressed in native C9 (26). The results are expressed as complement arbitrary units (CAU) per mL as defined by Standard #2 (25).

### Activation of platelets and neutrophils, and release of cytokines

Platelet β-thromboglobulin (BTG) (Human CXCL7/NAP-2 DuoSet ELISA, R&D Systems, Minneapolis, MO) and neutrophil myeloperoxidase (MPO) (Human Myeloperoxidase DuoSet ELISA, R&D Systems) were measured by ELISA and performed according to the manufacturer’s instructions. Cytokines were analyzed using a Bio-plex Human Cytokine 27 multiplex assay (Bio-Rad Laboratories, Hercules, CA), according to the manufacturer’s instructions.

### Hematology and microbiology

Samples for hematology and microbiology were analyzed in the routine laboratories at Oslo University Hospital.

### Data analysis

Statistical analyses were performed with the Mann-Whitney U test and Kruskal-Wallis tests using GraphPad Prism 9.5.1 (San Diego, CA). Data are presented as medians with interquartile ranges or individual dots for all data. A p<0.05 was considered significant.

### Ethical considerations

Ethical approval was provided by Regional Committee for Medical and Health Research Ethics of South-East Norway (REK 285790). Informed and written consent was provided from patients before inclusion in the study.

## Results

### Study cohort and demographics

The patient cohort demographics are presented in **Table 1**. All 17 included patients had AML in remission at the start of the conditioning regimen (baseline). Fourteen patients were alive at the end of the 200-day observation period; 12 of them in complete remission. Patient comorbidities encompassed cardiac conditions, including one case with atrial fibrillation and another with previous mitral valve plasty; and autoimmune conditions, including vitiligo and diabetes mellitus type I. Bacteremia was detected in 47% of patients, with the causative agents being *Enterobacter, Streptococcus, Stenotrophomonas, E. coli, Klebsiella, S. capitis*, and fungi included including *C. glaberata* and *A. fumigatus.* Eight patients developed acute GVHD and one of these developed chronic GVHD.

**Table 1:** Demographic data, comorbidities, complications and outcome.

Pretransplant patient characteristics	
**Included patients (n)**	17
**AML^1^ diagnosis (n**)	17
Gender (n)	
Male	11 (65%)
Female	6 (35%)
**Age **(median and range)	59 (25–73)
Male	51 (25-66)
Female	68 (53-73)
**BMI^2^ in Kg/m^2 **(median and range)	26 (19-31)
Male	26 (19-31)
Female	24 (21-30)
Comorbidities	
Cardiac	2 (13%)
Autoimmune	3 (18%)
Neurological	0 (0%)
Posttransplant outcomes	
Acute GVHD grad III-IV^3^	
Yes	8 (47%)
No	9 (53%)
Engraftment	
Days to ANC^5^ 0.5x10^9^/L	DAYS
Days to PLT^6^ 20 x10^9^/L	DAYS
Bacteremia	
Yes	8 (47%)
No	9 (53%)
EBV or CMV reactivation	
Yes	7 (41%)
No	10 (59%)
**Disease status at day 200** Remission	12 (86%)
Relapse	2 (14%)
**Outcome at day 200** Alive Dead	14 (82%) 3 (18%)

^1^ AML, acute myeloid leukemia; ^2^ BMI, Body Mass Index; ^3^ GVHD, graft versus host disease. ^4^ANC, neutrophils; ^5^PLT, platelets.

### Changes in cell populations, activation of neutrophils and platelets, and acute phase activation in patients undergoing HSCT

**Figure 1:**
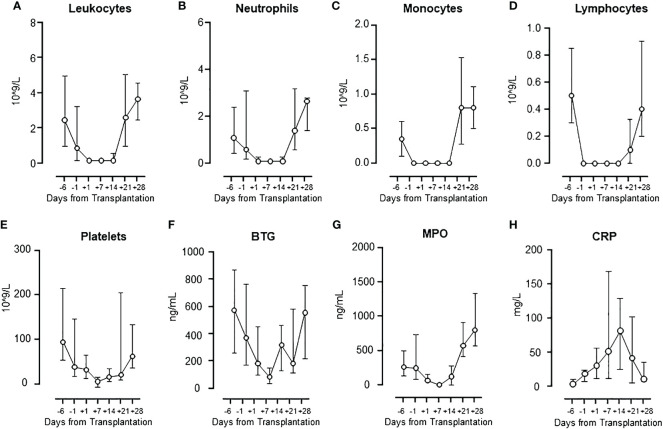
Cell population numbers and activation markers during HSCT. The changes in cell population numbers of leukocytes **(A)**, neutrophils **(B)**, monocytes **(C)**, lymphocytes **(D)**, and platelets **(E)**, and the levels of activation markers BTG **(F)** and MPO **(G)**, and CRP **(H)** are presented. Graphs show median ± interquartile range (n=17).

As expected, all patients experienced a marked and steady fall in all cellular components from baseline to lowest value, observed approximately one-week post-transplant (**Figures 1A–E**), followed by a subsequent increase to baseline levels or even higher after four weeks. At four weeks post-transplant all patients had achieved engraftment with full donor chimerism.

BTG, released from the platelets, showed a pattern virtually identical to the platelet count (**Figure 1F**), with a significant correlation (Spearman, rho=0.79, p=0.048) (**Supplementary Figure 1A**). MPO, released mainly from the neutrophils, showed a pattern virtually identical to the neutrophil count (**Figure 1G**), with a significant correlation (Spearman, rho=0.96, p=0.002) (**Supplementaryay Figure 1B**). C-reactive protein (CRP) increased steadily from baseline to a peak at one-week post-transplant, returning to baseline at day +28, reflecting the substantial acute phase reaction occurring during the early transplantation (**Figure 1H**). CRP and IL-6 correlated significantly (Spearman, rho=0.85, p=0.02).

### Total complement activity and activation product sC5b-9 in patients undergoing HSCT

**Figure 2:**
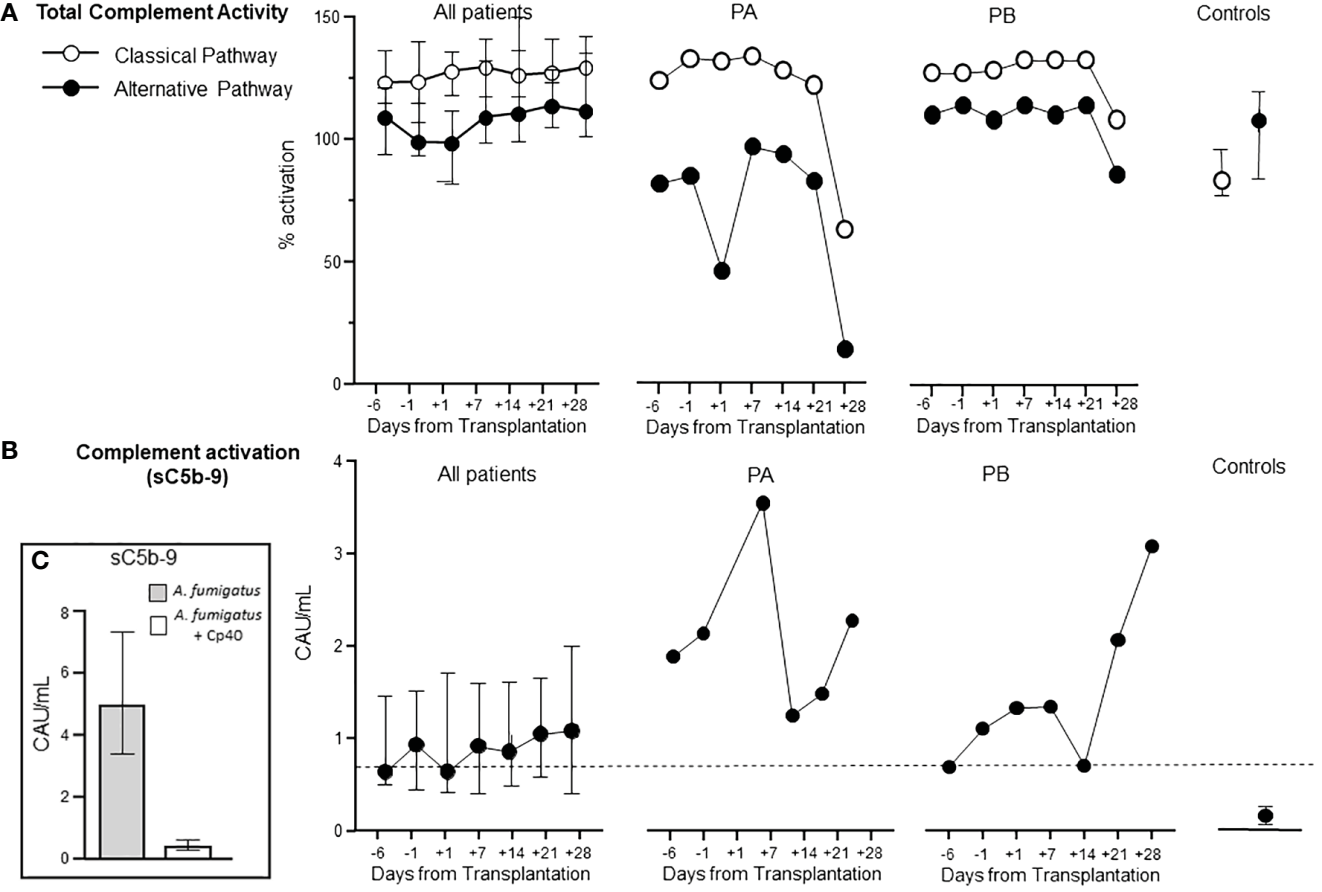
Complement function and activation during HSCT. The total functional complement activity of the classical and alternative pathway was measured using immunoassay (Wieslab®) with 100% as average reference normal value in all patients **(A**, left**)**. The degree of complement activation *in vivo* was measured as sC5b-9 in all patients **(B** left**)**. Two patients (PA and PB) showed clearly different patterns from the rest of the group for complement activity **(A** middle and right**)** and for complement activation **(B** middle and right**)**. Healthy controls are shown to the right in **(A, B)**. Graphs show median ± interquartile range (n=17). Dotted line in **(B)** represent upper reference value from normal blood donors. Panel **(C)** describes the effect of complement inhibition induced by *A. Fumigatus* - see below.

In contrast to the well-studied immune cell changes during HSCT, the complement function has been far less studied. Thus, we investigated whether the complement system was affected, either by being suppressed with reduced functional activity using the WIELISA test or being pathologically activated using the sC5b-9 test (**Figure 2**). Notably, we found that the complement function was completely preserved during the whole period, showing at least 100% function (mean of normal healthy controls) of both the classical and the alternative pathway (**Figure 2A**, left panel). Two of the patients, however, diverged substantially from the others. Patient A (PA) and patient B (PB) showed a substantial fall in complement activity in both pathways (**Figure 2A**, middle and right panel).

sC5b-9 levels were slightly elevated in the patients from admittance to the end of the study (**Figure 2B**, left panel). PA and PB, however, showed markedly increased complement activation (**Figure 2B**, middle and right panel). PB had a marked increase in sC5b-9 already at admittance, peaking at day +7, then declining and reaching a new top at day +28. The patient suffered severe complications diagnosed at day +24 as sinusoidal obstruction syndrome with high transaminases (AST 1876, ALT 1257), required dialysis, developed GVHD and TA-TMA, and died at day +105. PB had a sC5b-9 pattern similar to PA, reaching the highest level at day +28. This patient suffered from infection diagnosed as *S. maltophilia* at day +14, followed by *S. capitis* and *C. glaberata* at day +19 and *A. fumigatus* in the lungs at +day 30. The patient died at day +90 from relapse of AML. The sC5b-9 increase in these two patients is consistent with the fall in functional activity described above, consistent with consumption of the native components. PA and PB were two of the three patients that died during the 200 days observation period. A third patient died already on day +11 due to septic shock and was not included due to lack of samples. To further characterize the differences between PA/PB and the other patients with uneventful course, the CRP and IL-6 levels were compared (**Supplementary Figure 2**). A higher level of both CRP (**Supplementary Figure 2A**) and IL-6 (**Supplementary Figure 2B**) was found in the PA/PB patients, with a substantial increase during the course, as compared to the other patients.

### Comparisons between plasma cytokines from AML patients pre-HSCT and healthy controls

**Figure 3:**
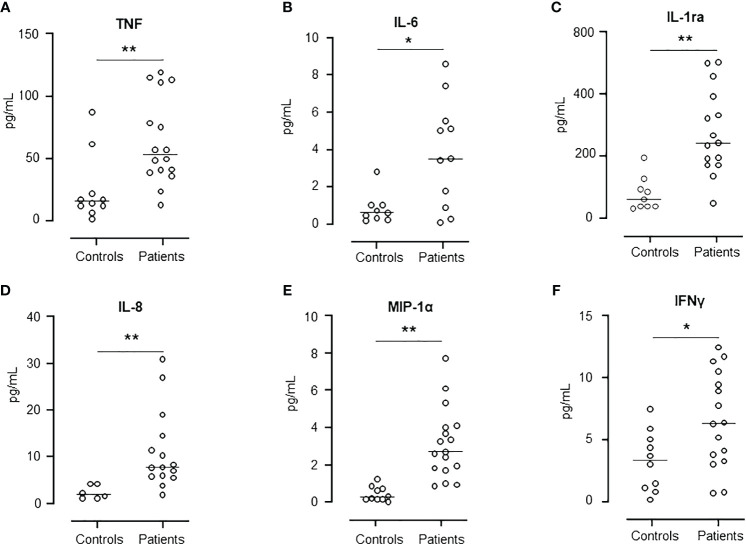
Comparison of cytokine release levels between patients at day -6 and healthy controls. Figure shows the levels of TNF **(A)**, IL-6 **(B)**, IL-1ra **(C)**, IL-8 **(D)**, MIP-1α **(E)**, and IFNγ **(F)**. Graph shows median and all values, n=10 in healthy control group and n=17 in patient group. Statistical analysis was done using the Mann-Whitney U test. Significance is shown as *p < 0.05, **p < 0.01.

Six cytokines representing interleukins, chemokines and interferons were selected for comparison between AML patients at hospital admission and healthy controls (**Figure 3**). TNF, IL-6, IL-1ra, IL-8/CXCL8, MIP-1α/CCL3 and IFNγ (**Figures 3A–F**) were all statistical significantly higher in the patients than in the healthy controls, reflecting the hyperinflammatory state in AML patients.

### Patterns of plasma cytokine levels in patients undergoing HSCT

**Figure 4:**
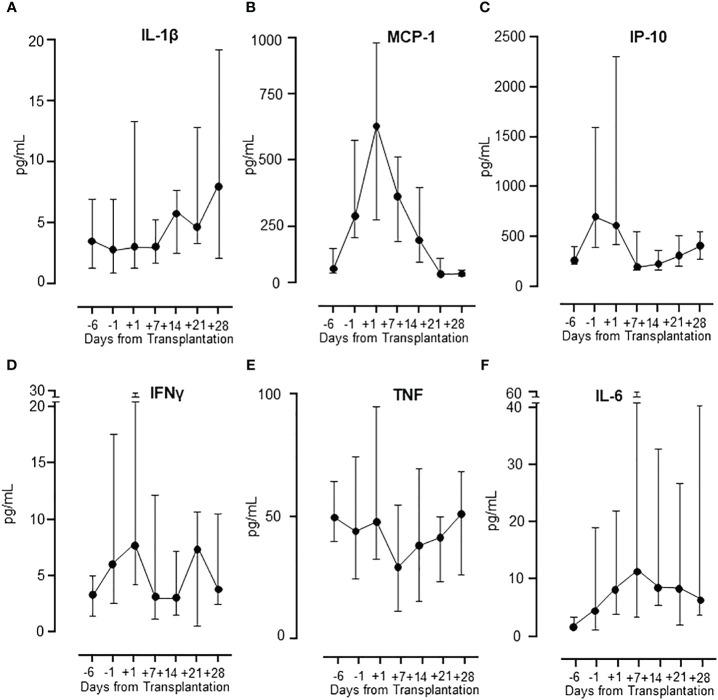
Cytokine release in patients during HSCT. The different patterns of cytokine release in patients during the whole observation period are illustrated by IL-1β **(A)**, MCP-1 and IP-10 **(B, C)**, IFNγ **(D)** and TNF and IL-6 **(E, F)**. Graphs show median ± interquartile range (n=17).

The concentration of several cytokines was measured in the patients during the whole period (**Figure 4**). Three different patterns were observed. Pattern 1, a gradual increase in cytokine release during the post-transplant period was seen for IL-1β and IL-13, illustrated by IL-1β (**Figure 4A**). Pattern 2, a steep increase from baseline to a peak at transplantation, eventually declining to baseline levels, was seen for MCP-1/CCL2, IP-10/CXCL10, IL-8/CXCL8, MIP-1α/CCL3, G-CSF and FGF-basic, illustrated by MCP-1/CCL2 (**Figure 4B**) and IP-10/CXCL10 (**Figure 4C**). Pattern 3, a combination of pattern 1 and 2, showing a distinct peak at transplantation, decreasing to baseline followed by a subsequent second increase two weeks after transplantation, was seen for IFNγ and eotaxin, illustrated by IFNγ (**Figure 4D**). The remaining eight cytokines displayed differential patterns with TNF, IL-1ra, IL-9, MIP-1β/CCL4 and RANTES/CCL5 showing a distinct decline at day +7, illustrated by TNF (**Figure 4E**), and an opposite increase at day +7 seen for IL-6 (**Figure 4F**).

### Patterns of cytokine levels in HSCT patient incubated with *E. coli*

**Figure 5:**
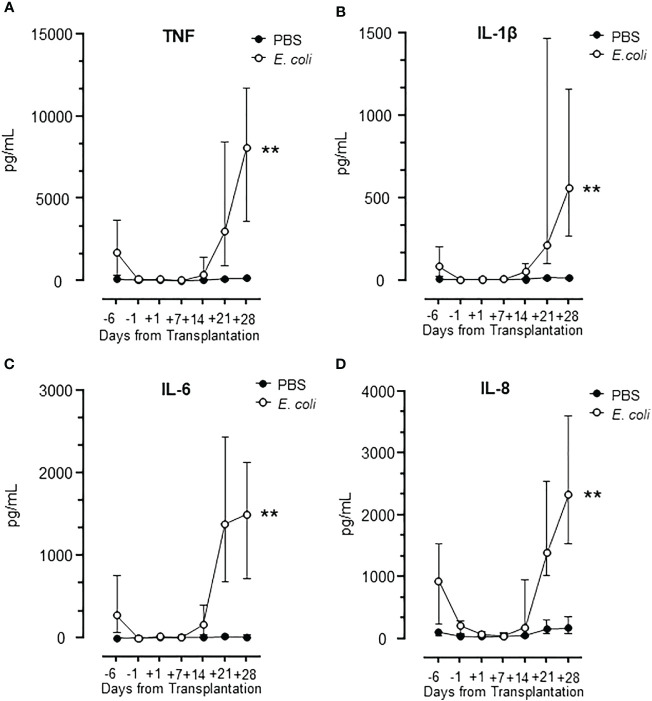
Cytokine release during HSCT in lepirudin whole blood incubated with *E. coli*. The pattern of cytokines TNF **(A)**, IL-1β **(B)**, IL-6 **(C)**, and IL-8 **(D)** are shown in samples obtained during the whole observation period. Graphs show median ± interquartile range (n=17). Statistical analysis was done using the Mann-Whitney U test. Significance is shown as: **p < 0.01.

Whole blood samples from the observation period were incubated with *E. coli* to induce a robust cytokine release with PBS as negative control (**Figure 5**). Four classical proinflammatory cytokines were analyzed: TNF, IL-1β, IL-6 and IL-8/CXCL8 (**Figures 5A–D**). The results showed a virtually identical pattern for all cytokines with a substantial response to *E. coli* at admittance, no response from day -1 to day +7, and then an abrupt increase in response from day +14 (**Figure 5**). The cytokine response followed the leukocyte count closely (**Figure 1**).

Notably, the expression of TNF, IL-1β, IL-6, and IL-8/CXCL8 following incubation with *E. coli* was substantially and significantly (p<0.01 for all) elevated at day +28 compared to day -6 (hospital admission) (**Figure 5**), consistent with a more potent inflammatory response from the donor cells after the engraftment compared to the cells from the patient at admission.

### Comparisons of the cytokine response following challenge with PAMPs and DAMPs to whole blood from AML patients pre-HSCT as compared to healthy controls

**Figure 6:**
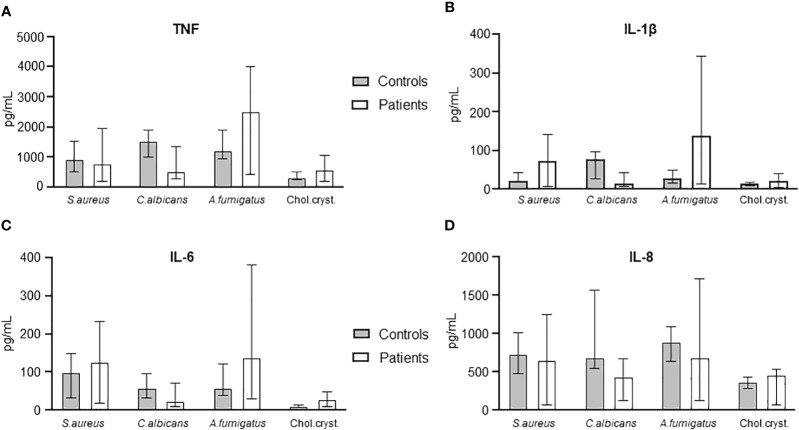
Comparison of cytokine levels in *S. aureus, C. albicans, A. fumigatus*, and cholesterol crystals between HSCT patients at day -6 and healthy controls. TNF **(A)**, IL-1β **(B)**, IL-6 **(C)**, and IL-8 **(D)** are shown compared between controls and patients. Graphs show median ± interquartile range (n=6). Statistical analysis was done using the Mann-Whitney U test.

We then compared the patients’ blood at admission with healthy controls with respect to cytokine release (TNF, IL-1β, IL-6, and IL-8/CXCL8) in response to PAMPs and DAMPs relevant for immunosuppressed patients, i.e. *S. aureus*, *C. albicans, A. fumigatus*, and cholesterol crystals (**Figure 6**). No statistical differences were seen between patients and health controls.

### Patterns of cytokine levels in whole blood from patients undergoing HSCT when incubated with PAMPs and DAMPs

**Figure 7:**
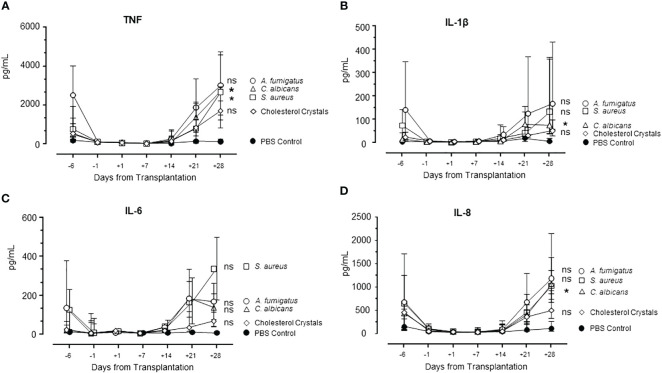
Cytokine release during HSCT in *S. aureus, C. albicans, A. fumigatus*, and cholesterol crystals incubated in whole blood. Pattern of cytokines TNF **(A)**, IL-1β **(B)**, IL-6 **(C)**, and IL-8 **(D)** during the whole observation period of patients are shown. Graphs show median ± interquartile range (n=6). Statistical analysis was done using Mann-Whitney U test. Significance shown as: ns for p >0.05, *p < 0.05.

The pattern of cytokine release was similar for TNF, IL-1β, IL-6, and IL-8/CXCL8 (**Figures 7A–D**), declining from day -6 to close to zero and markedly increasing from day +14, reaching levels at day +28 comparable to or higher than those at day -6. Statistically significant higher levels at day +28 compared to day -6 were seen for TNF incubated with *S. aureus*, and for TNF, IL-1β and IL-8 incubated with *C. albicans.*

### Effect of complement inhibition on the expression of cytokines during HSCT

**Figure 8:**
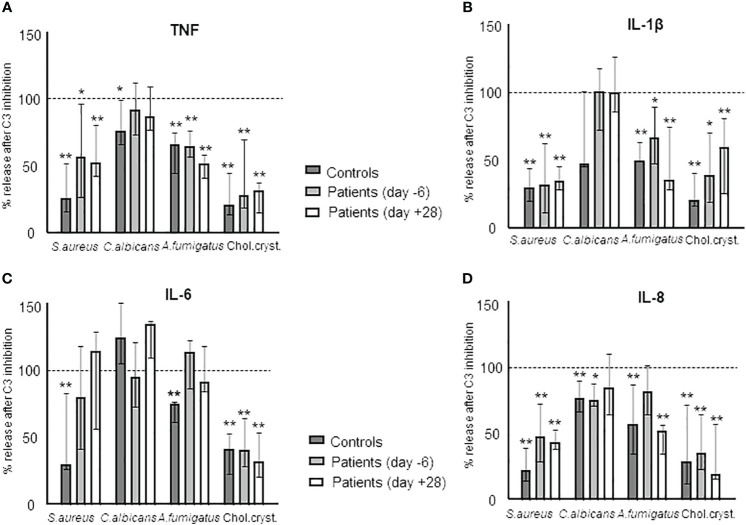
The complement dependency of the cytokine release during HSCT in samples obtained from HSCT patients *in vitro* incubated with *S. aureus, C. albicans, A. fumigatus*, and cholesterol crystals. Sample comparison is between samples incubated with the respective agents and sample pre-treated with the C3-inhibitor CP40 and then incubated with the respective agent. The effect is illustrated by the degree of inhibition in % as compared to the non-inhibited sample: TNF **(A)**, IL-1β **(B)**, IL-6 **(C)**, and IL-8 **(D)**. Graph shows median ± interquartile range (n=6). Statistical analysis was done using the Kruskal-Wallis test. Significance shown as: *p < 0.05, **p < 0.01.

Finally, we investigated the role of complement on the cytokine response using the C3 inhibitor Cp40 to determine complement-dependent responses. First, we documented that Cp40 completely blocked the formation of sC5b-9 in blood incubated with *A. fumigatus* (**Figure 2C**). We then incubated blood from healthy controls and patients at day -6 and day +28 with *S. aureus*, *C. albicans*, *A. fumigatus*, and cholesterol crystals (**Figure 8**). The results are shown as % change of cytokine release in the samples pre-treated with Cp40 compared to PBS and significance is calculated as difference between the uninhibited sample and the Cp40 pre-inhibited sample as determined by Kruskal-Wallis. Overall, the results were similar in healthy control and patient samples, consistent with the intact complement function during HSCT. There was a statistically significant inhibition caused by Cp40 for TNF and IL-1β release induced by *S. aureus*, *A. fumigatus*, and cholesterol crystals (**Figures 8A, B**); for IL-6 release induced by cholesterol crystals (**Figure 8C**); and for IL-8 release for all the activators (**Figure 8D**).

## Discussion

This study presents novel insight into the innate immune system during HSCT. We observed that the complement system remained functionally intact during the whole period in patients with uneventful treatment courses and that the complement activity did not differ compared to an age- and sex-matched cohort. This indicates that the complement system can be activated in the same manner as in healthy individuals to protect against infection and can induce adverse inflammatory responses if activated improperly.

How the complement system changes during the early phase after transplantation is inadequately explored. Complement proteins are mainly produced in the liver and extensively consumed during infectious challenges. Despite the liver cell stress caused by conditioning regimens (27), alloreactivity, GVHD prophylaxis, antifungal azoles, and persistent systemic inflammation caused by mucous membrane inflammation and bacterial translocation, most patients maintained a fully active complement system, protecting them against infection. However, certain aspects of complement functionality, such as phagocytosis, will be affected by the lack of effector cells during HSCT. Other complement functions, such as lysis of bacteria through the terminal complement complex will be intact and of particular importance for protection against complement sensitive strains. Furthermore, an intact complement activity will be of importance when treating patients with immunotherapy that partly rely on complement for lysing cancer cells. On the other hand, an intact complement system may be a threaten to the patients by causing tissue damage through over-activation occurring during complications like TA-TMA and GVHD.

An important question is to which degree the complement system is activated during the early post-transplant period. Our data indicate that neither the conditioning regime, GVHD prophylaxis, stem cell infusion, nor the early post-transplant toxicity significantly alters the complement system. Baseline sC5b-9 levels in patients were slightly elevated compared to controls, aligning with the modest increase in inflammatory response observed in cytokines. This modest complement activation at baseline did not appear to contribute to a higher degree of post-transplant complement activation. The complement system was not investigated in the donors for logistical reasons. However, complement deficiencies in the normal population is extreme rare (28) and the use of non-parametric data handling would not have been influenced by a single deficiency.

A distinct activation pattern was observed in two patients (PA and PB), both of whom encountered severe complications and died during the observation period. Both patients showed a similar pattern of complement activation as detected by sC5b-9 with an initial increase followed by a decline and then an escalating increase until day +28. This final increase correlated with an abrupt decrease in the complement function in both classical and alternative pathway, which is consistent with a consumption of the native component during activation. Retained complement function both of classical and lectin pathway was observed in all patients with uneventful course. This is of particular interest for the classical pathway, since C1q is one of few complement components that are not produced by the liver, but by immune cells, mainly the monocytic line (29, 30), which are virtually depleted during HSCT. However, C1q is also produced by non-immune sources such as osteoclasts (31) and endothelial cells (32). This could explain why the classical pathway remained fully active, together with the fact that a certain drop in C1q concentration would not lead to lower classical pathway activity since C2 is the rate limiting factor (33).

Patients PA and PB illustrate how complement can be activated with the same pattern despite strictly different etiology: PA displayed DAMP-induced, sterile inflammation due to sinusoidal obstruction syndrome and GVHD, while PB suffered from PAMP-induced infectious inflammation due to bacterial and fungal infection. Both PA and PB showed a marked increase in CRP and IL-6 as compared to patients with uneventful course. These results indicate that systemic complement activation is involved in the pathogenesis of different complications leading to severe morbidity during HSCT, which is in accordance with a previous study (19). Recent results indicate that complement inhibition is beneficial in pediatric TA-TMA (18) indicating that complement may play a role in developing HSCT-related complications.

The activation markers BTG and MPO, indicators of platelet (34) and neutrophil (35) activation, followed a pattern virtually identical to the cell counts of the individual cell populations. Accordingly, there was a highly significant correlation between platelet counts and BTG release, and between neutrophil counts and MPO, supporting the respective cell types to be the main source of these mediators. In contrast to BTG and MPO, the systemic marker of inflammation CRP showed a different pattern (36) with normal levels at baseline that increased substantially to peak at day +7, followed by a decrease to baseline. IL-6 has been shown to be the main promotor of CRP (37). The strong correlation between IL-6 and CRP found in this is consistent with previously published data in HSCT patients (38).

As we observed a general increase in cytokines in whole blood at day -6 compared to healthy controls, we examined the level of all cytokines included in the study throughout the observation period. Three distinct patterns were observed. *First*, IL-1β and IL-13 were low at baseline and increased one to two weeks after transplantation, reaching a maximum level at days +21 and +28. *Second*, six cytokines, of which comprised four chemokines, showed a distinct increase from the baseline, reaching a peak at transplantation and declining thereafter to baseline. MCP-1/CCL2, IP-10/CXCL10, IL-8/CXCL8, MIP-1α/CCL3, G-CSF and FGF-basic belonged to this group. This second pattern reflects an interesting hyperinflammatory response before and during the aplastic phase of HSCT and suggests that these cytokines are produced extra-vascularly. This has been shown for MCP-1 which can be produced by endothelial cells, epithelial cells and smooth muscle cells (39), and IP-10/CXCL10 produced by endothelial cells, epithelial cells, and keratinocytes (40). Both MCP-1/CCL2 and IP-10/CXCL10 are released in response to IFNγ signaling (40, 41). *Third*, IFNγ and eotaxin showed two distinct peaks, corresponding to the peak at transplantation seen for pattern two and a late increase seen for pattern one, suggesting a contribution from extravascular cells and donor leukocytes. IFNγ is a significant mediator of the immune response and has previously been shown to be released by T-cells, NK cells, NKT cells, macrophages, dendritic cells, and B cells (42, 43).

Considering the heightened susceptibility to infections in the patients undergoing allogeneic HSCT, we aimed to delve deeper into the characterization of cytokine release by incubating their blood with *E. coli* (20). The classical proinflammatory cytokines TNF, IL-1β, IL-6 and IL-8/CXCL8, display a typical response in normal blood incubated with *E. coli* (44). Blood from the HSCT patients at baseline responded to *E. coli* with a similar pattern as described for blood from heathy donors, whereas this response declined to undetectable levels during the aplastic phase. A post-transplant release of the cytokines was markedly increased at day +28 compared to pre-transplant indicating a more robust response to *E. coli* in the transplanted cells. The elevated cytokine levels seen at day +28 in the patients may be related to trained immunity, a concept suggesting a memory-like state in innate immune responses (45, 46). This could result from prolonged exposure to DAMPs and PAMPs during the early engraftment phase, when gastrointestinal barrier breach secondary to mucositis remains prominent.

To determine if this cytokine response was *E. coli* specific, blood was incubated with other PAMPs and DAMPs commonly observed during the early post-transplant period such as *S. aureus*, *C. albicans* and *A. fumigatus* (47). Cholesterol crystals were included to the experimental setup to simulate DAMP-induced sterile inflammation, a crucial component of the prevailing clinical manifestation following HSCT and associated toxicity (48). Cholesterol crystals have also been shown to induce TA-TMA, a common complication post-HSCT (49). Our data indicate that the augmented inflammatory responses post-transplant is not *E. coli* or pathogen specific. Previous studies on the cytokine levels post-transplant have shown a similar trend with an increase in cytokine release (50, 51), and confirmed this to be an increase compared to levels observed prior to transplantation (51).

The final goal of this study was to determine the role of complement in the cytokine release in HSCT patients. The effect of complement inhibition, using a C3 inhibitor, differed between the different agents used. *S. aureus, A. fumigatus* and cholesterol crystal-induced cytokine release were more efficiently reduced by C3 inhibition than cytokines induced by *C. albicans.* Overall, the effect of complement inhibition was comparable in samples from patients and from healthy controls. These results indicate a retained complement-dependent effect in patients undergoing HSCT, which is efficiently blocked by a complement inhibitor with the same effects on the secondary induced cytokine release as observed in normal controls.

In conclusion, patients undergoing HSCT have a fully intact complement system, which is generally was not activated during the early post-transplant period. However, two patients who died due to opportunistic infection and TA-TMA, respectively, underwent a substantial complement activation. The cytokine inflammatory response studied *in vitro* was reduced by inhibiting complement activation. Thus, we provide novel insights into the innate immune responses during HSCT and new avenues for potential complement therapies during HSCT.

## Data availability statement

The original contributions presented in the study are included in the article/**Supplementary Material**. Further inquiries can be directed to the corresponding author.

## Ethics statement

The studies involving humans were approved by Ethical approval was provided by Regional Committee for Medical and Health Research Ethics of South-East Norway (REK 285790). Informed and written consent was provided from patients before inclusion in the study. The studies were conducted in accordance with the local legislation and institutional requirements. The participants provided their written informed consent to participate in this study.

## Author contributions

BF: Conceptualization, Investigation, Project administration, Writing – original draft, Writing – review & editing, Data curation, Formal analysis, Methodology. LC: Data curation, Formal analysis, Investigation, Methodology, Writing – review & editing. CS: Data curation, Formal analysis, Investigation, Methodology, Writing – review & editing. KM: Data curation, Formal analysis, Methodology, Writing – review & editing. CL: Data curation, Methodology, Writing – review & editing, Project administration. JH: Data curation, Methodology, Writing – review & editing, Formal analysis. TG-D: Writing – review & editing, Conceptualization, Project administration, Supervision. RW: Conceptualization, Project administration, Supervision, Writing – review & editing, Data curation. TE: Conceptualization, Data curation, Writing – review & editing, Methodology. GT: Conceptualization, Writing – review & editing, Investigation, Resources, Supervision. PG: Conceptualization, Investigation, Supervision, Writing – review & editing, Funding acquisition, Methodology. AB-D: Conceptualization, Investigation, Supervision, Writing – review & editing, Resources. TT: Conceptualization, Investigation, Supervision, Writing – review & editing, Methodology, Project administration. TM: Conceptualization, Investigation, Project administration, Supervision, Writing – review & editing, Funding acquisition, Resources, Validation, Visualization, Writing – original draft.
