# Supplementary material for: The function of the complement system remains fully intact throughout the course of allogeneic stem cell transplantation
Source: Front Immunol. 2024 Jun 13;15:1422370. doi: 10.3389/fimmu.2024.1422370 (PMC11208304; doi:10.3389/fimmu.2024.1422370)
Supplement: Supplementary file 1 [file DataSheet_1.pdf]

Supplementary Material

Suppl. Fig 1

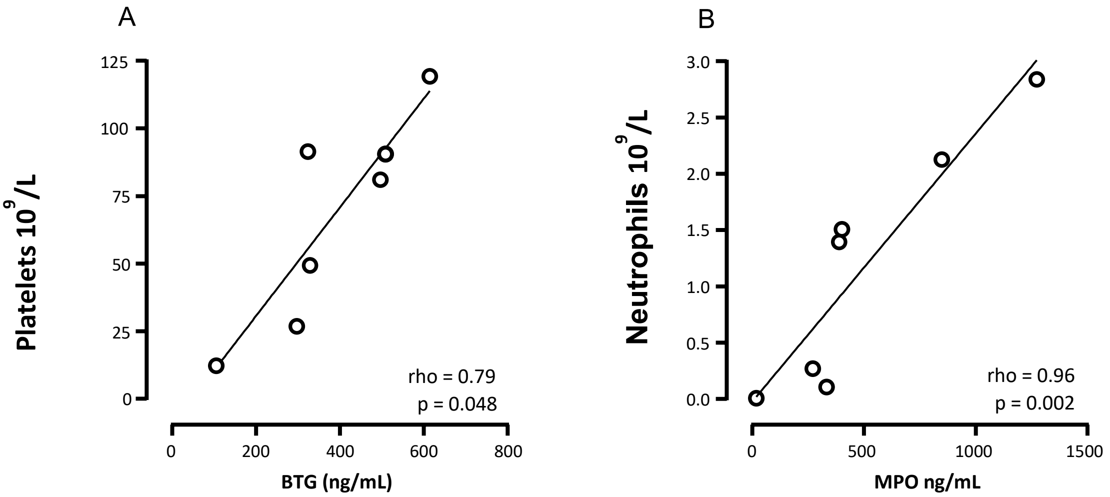

Suppl. Fig 1 Legend. Correlation between concentration of released proteins and the number of the of the parent cells. The correlation between BTG and platelets (A) and MPO and neutrophils (B) are presented. The plotted values are the mean of all collected patient samples (n=17) at the different collection time points.

Suppl. Fig 2

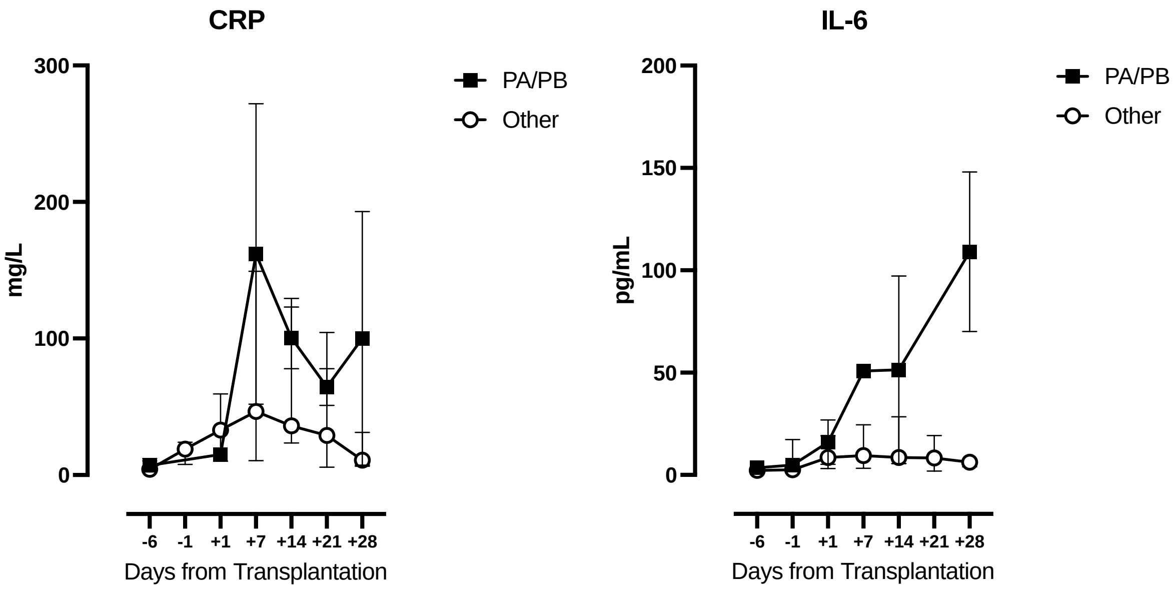

Suppl. Fig 2 Legend. CRP and IL-6 in patients A and B. The CRP and IL-6 values during the course of HSCT in the two patients (PA/PB) experiencing severe complications compared to the rest of the patients who had an uneventful course (Other).

Supplementary Table 1. An overview of conditioning regimens, graft source, and GVHD prophylaxis in the included patients.

Patient ID	Gender	Conditioning regime	Graft source	GVHD prophylaxis
#1	Male	Fludarabine/Treosulfan	PBSC	Methotrexate, Ciclosporin, ATG[1]
#2	Male	Fludarabine/Treosulfan	PBSC	Methotrexate, Ciclosporin, ATG
#3	Male	Fludarabine/Bulsulfane	PBSC	Methotrexate, Ciclosporin, ATG
#4	Female	Fludarabine/Treosulfan	PBSC	PT-Cy, MMF, Ciclosporin
#5	Female	Fludarabine/Treosulfan	PBSC	Methotrexate, Ciclosporin, ATG
#6	Female	Fludarabine/Treosulfan	PBSC	Methotrexate, Ciclosporin, ATG
#7	Male	Fludarabine/Treosulfan	PBSC	Methotrexate, Ciclosporin, ATG
#8	Male	Fudarabine/Busulfane/Thiotepa	PBSC	PT-Cy, MMF, Ciclosporin
#9	Female	Fludarabine/Treosulfan	PBSC	Methotrexate, Ciclosporin, ATG
#10	Male	Fludarabine/Treosulfan	PBSC	PT-Cy, MMF, Ciclosporin
#11	Male	Fludarabine Bulsulfane	PBSC	PT-Cy, MMF, Ciclosporin
#12	Female	Fludarabine/Treosulfan	PBSC	Methotrexate, Ciclosporin, ATG
#13	Male	Fludarabine/Treosulfan	PBSC	Methotrexate, Ciclosporin, ATG
#14	Male	Fludarabine/Bulsulfane	PBSC	Methotrexate, Ciclosporin, ATG
#15	Female	Fludarabine/Bulsulfane	PBSC	Methotrexate, Ciclosporin, ATG
#16	Male	Fudarabine/Busulfane/Thiotepa	BM	PT-Cy, MMF, Ciclosporin
# 17	Male	Fludarabine/Treosulfan	PBSC	Methotrexate, Ciclosporin, ATG

Abbreviations**:** ATG: Anti-thymocyte globulin, BM: Bone marrow, PBSC: Peripheral blood stem cell, MMF: mycophenolate mofetil, PT-Cy: post-transplant cyclophosphamide.
